# 现代医学事业发展的新理念——充分利用互联网、大数据、人工智能

**DOI:** 10.3779/j.issn.1009-3419.2018.03.02

**Published:** 2018-03-20

**Authors:** 逊 张

**Affiliations:** 300051 天津，天津市胸科医院 Tianjin Toracic Hospital, Tianjin 300051, China

**Keywords:** 医学, 互联网, 大数据, 人工智能, Medicine, Internet, Large data, Artifcial intelligence

中共十九大提出要建设网络强国、数据中国、智慧社会、推动互联网、大数据、人工智能和实体经济深度融合，这已经成为党和国家的最高战略。我们面临着如何充分利用互联网、大数据与人工智能促进我国医疗事业迅速发展的新问题。我们需要新理念、新技术、新方法，与时俱进，引领时代的发展。

互联网医疗未来的发展趋势可以概括为以下几个方面：人机互动(医疗检测设备与医院或医生的对接)、方便患者就医(预约挂号、寻医问药)、信息检测(医院对内的HIS系统、对外的互联网舆情检测)、医疗数据的整理与开发(利用互联网进行数据库的建设)、出院患者管理系统(利用互联网进行医患沟通)以及建立同行医生之间互相沟通的平台(利用互联网进行远程会诊)等。

医疗大数据涵盖的内容很多，其中基于电子病历的临床数据是最核心的医疗数据，也是最有科研价值的数据。电子病历的定义可以分为狭义和广义两种：狭义的电子病历仅是纸质病历的电子化；广义的电子病历是指医务人员在医疗活动过程中形成的文字、符号、图表、影像、切片等资料的总和，涉及患者信息的采集、存储、传输、处理和利用的所有过程信息。全集成、全过程、全周期、智能化和多视图是电子病历共有的五大特征。国家卫计委对我国电子病历的系统功能应用水平，制定了一套完整的评级标准，共分为8个等级(0级-7级)，等级越高表示电子病历系统的水平越高。根据2015年中华医院信息网络大会的统计，参与数据填报的2, 622家医院，其中电子病历应用水平达到5级及以上的医院仅有6家，占比为0.6%。显示我国医院电子病历系统应用水平还非常低，需要大力改进和提高。基于电子病历的医疗大数据互联互通，对政府、对医院、对医生、对患者、对药企、对保险这六大核心要素都提供了重要支撑，称之为医疗行业的基础设施建设。由此不难理解国家为什么高度重视医疗互联网大数据的建设。

医疗大数据不仅仅指数据量大，还包括医疗数据的复杂性和多维度，涉及到影像、病理、分子诊断、各种治疗方法等多学科信息。目前医疗机构之间，甚至同一家医院不同专业科室之间的信息壁垒，导致大量的临床相关数据无法集中采集、存储、分析，从而被束之高阁无法使用。因此，虽然我国每年诊治了上亿人次的患者，积累了世界上最多的临床医疗数据，但是在制定各种疾病的国际诊疗指南时，很少采纳中国的医疗数据，就是因为我们的医疗数据是分散的、不标准的、非结构化的，因而是无法采用的。为此，我们需要从医院电子病历的标准化、结构化等基础性建设入手，建立安全、有效的互联网数据库，将医疗大数据的价值充分地发挥出来。

我国胸外科专业自2014年开始，采用标准统一、各自独立的原则，在第三方互联网医疗大数据平台(零氪科技)的大力支持下，建设了符合中国国情的互联网数据库，迄今已经有200多家三级综合医院和专科医院的胸外科加入其中，结构化和高随访率的胸部肿瘤病历已经累积了几十万例，成为国际上数量最大的胸部肿瘤数据库。通过对数据库相关数据的分析研究，目前已经产生了一批高质量、多中心、大数据的临床研究成果。随着胸外科互联网数据库的广泛使用，必将有更多、更好的临床研究成果问世，对提高我国胸外科在国际上的影响力，增大对国际胸部肿瘤疾病诊疗指南修订的话语权，必将发挥越来越大的作用。

医疗术语的标准化是建设好数据库、开展临床多中心研究的基础。我国目前各个临床专业的数语都缺少标准化。国务院办公厅于2017年5月发布的《深入医药卫生体制改革2017年重点工作任务的通知》中，明确提出要"加快推进医学名词术语的统一"。在这方面，胸外科专业走在了其他临床专业的前面。2016年中国医师协会胸外科医师分会成立了"胸外科专业术语标准化委员会"，集中了全国各地胸外科专家的意见，经过反复讨论，制定了《胸外科疾病标准化诊疗术语》，并于2017年4月由人民卫生出版社出版发行。成为我国第一个制定了本专业疾病标准化诊疗术语的专业，这是一项具有里程碑意义的工作。

制定了胸外科疾病标准化诊疗术语，仅是向高标准数据库建设迈出的第一步，如何在临床工作中落实还有大量艰苦的工作要做。中国医师协会胸外科医师分会于2018年1月在厦门大学第一附属医院召开了"胸外科标准化、结构化电子病历现场推广会"。学习和推广厦门大学第一医院的先进经验。相信随着胸外科标准化、结构化电子病历的不断完善和推广使用，我国胸外科专业的电子病历水平一定会有一个质的飞跃!

人工智能在胸外科的应用前景，目前日趋成熟的包括人工智能在医疗数据库建设(医疗数据采集、录入、存储和数据统计分析)中的应用、在辅助胸部小结节影像诊断中的应用、在辅助胸部肿瘤患者最佳治疗方案选择中的应用以及在辅助胸部肿瘤病理诊断中的应用等方面。

由于每日都有大量的临床医疗数据传输到数据库，其数量还在与日俱增，这种海量的医疗数据仅仅依靠人工输录是无法完成的。人工智能则发挥了非常重要的作用。通过机器学习，(零氪科技)互联网数据库形成了4, 000多个病历阅读规则、60, 000多个病历阅读字典，将不同地区、不同医院、不同医生在病历中的描述性语言(包括一些方言和医生的习惯用语)结构化为机器可以识别的语言。经人工智能自动处理后录入数据库的病历数量占了病历输录总量的80%以上，而且这一比例还在不断提高。与人工输录相比较，人工智能输录临床数据，不仅速度提高了近10倍，输录质量提高了20倍，而且输录数据的准确率达到了99%以上。人工智能已经成为建设优质互联网医疗数据库不可缺少的工具。

人工智能辅助肺部小结节影像诊断已经日趋成熟。2017年，由零氪科技、阿里云和英特尔三家公司联合举办的"人工智能在肺部小结节诊断中的应用全球比赛"，吸引了来自全球20个国家和地区、2, 887个团队、3, 953名选手参加的历时半年的比赛，成为全球同类项目中参加人数最多、影像病历数量最大、历时时间最长的人工智能比赛，有力地促进了人工智能在辅助肺部小结节影像诊断中的应用。目前，人工智能辅助肺部小结节影像诊断，已经不仅仅局限于肺部小结节的定位(即有无小结节)，而且可以对肺部小结节的病理性质(良性或恶性)进行辅助定性诊断。人工智能辅助影像诊断的临床应用，将大大提高我国基层医院胸部影像的诊断水平，促进区域性的人工智能辅助胸部影像诊断中心的形成，从而推进我国分级诊疗工作的开展。

随着人工智能在临床的广泛应用，必将促进临床医疗水平的不断提高，大大缩小不同地区、不同医院之间医疗水平的差异，提高同质化医疗水平，从而推动我国医疗事业的快速发展。但是无论人工智能多么发展，人工智能的作用只是辅助医生，而不是替代医生。作为医生，我们应当热情欢迎人工智能在临床的应用和普及，让人工智能最大限度地辅助医者、普惠患者。

